# Avolition Characterizes the Chronic Fatigue Experienced in Quiescent Inflammatory Bowel Disease

**DOI:** 10.3390/biomedicines13010125

**Published:** 2025-01-07

**Authors:** Tristan Gabriel-Segard, Margherita Boltri, Mathilde Barrau, Catherine Massoubre, Stéphane Paul, Xavier Roblin

**Affiliations:** 1 Service de Psychiatrie Transversale, Centre Hospitalo-Universitaire de Saint Etienne, Hôpital Nord, 42055 Saint Etienne, France; 2 CIRI—Centre International de Recherche en Infectiologie, Team GIMAP, Univ Lyon, Université Claude Bernard Lyon 1, Inserm, U1111, CNRS, UMR530, CIC 1408 Vaccinology, 42023 Saint Etienne, France; 3 I.R.C.C.S. Istituto Auxologico Italiano, Experimental Laboratory for Metabolic Neurosciences Research, 28824 Piancavallo, Italy; 4 Service de Gastroentérologie, Centre Hospitalo-Universitaire de Saint Etienne, Hôpital Nord, 42055 Saint Etienne Cedex 2, France

**Keywords:** inflammatory bowel disease, fatigue, avolition, negative syndrome, motivation

## Abstract

**Background and Aims:** Avolition is a symptom responsible for a high burden in patients suffering from psychiatric diseases. It refers to a motivation loss for initiating and maintaining goal-directed activities, often called fatigue by patients. Fatigue is a widespread complaint of patients suffering from inflammatory bowel disease (IBD), significantly impacting patients’ well-being, even during the quiescent stage of the disease. We here address the hypothesis that fatigue experienced by IBD patients is associated with motivational impairment. **Methods:** Patients presenting IBD (n = 110) in a quiescent stage of Crohn’s disease (CD) (n = 60) and ulcerative colitis (UC) (n = 50) were enrolled and classified following their declared experience of fatigue (n = 58) or not (n = 52). Patients were phenotyped using self-administered scales for fatigue experience, bowel disease disability, quality of life and mental health symptoms. **Results:** The self-administered negative symptoms scale scores identified avolition as a specific feature of fatigue experience: fatigued vs. no-fatigue in the CD group (3.806 vs. 2.103; *p* = 0.003) and in the UC group (2.815 vs. 1.174; *p* = 0.003). This difference is independent of current depressive disorder and previous history of depressive disorder. Avolition associates and correlates with the experience of fatigue (r = 0.49) in multivariate analysis. **Conclusions:** To tackle the question of fatigue in IBD, research should consider investigating the biological mechanisms implicating intestinal physiopathology of IBD in the impairment of brain structure involved in motivation. This may open new fields for treatment in targeting structures of the brain reward system.

## 1. Introduction

Inflammatory bowel diseases (IBD) are chronic diseases that include Crohn’s disease (CD) and ulcerative colitis (UC). Digestive symptoms significantly impact the patient’s quality of life, and fatigue is the most prevalent extra-intestinal symptom for patients [1]. The prevalence of fatigue concerns 72% of patients with an active disease and 47% of patients with a remitted state of IBD [2]. A study report detailed the dimension of fatigue using the Multidimensional Fatigue Inventory (MFI), with 31.5% of patients reporting physical fatigue, 18% of mental fatigue, 14.5% of reduced motivation, and 25.4% of reduced activity [3]. Fatigue is reported as a factor associated with low quality of life, long duration of the disease, and symptoms of anxiety in the stage of remission of the disease [4]. Some pathophysiology hypotheses are proposed to understand the setting of fatigue and its persistence in IBD conditions. Massive inflammatory mediators released during active disease flare-ups are linked to fatigue experience [5]. During the quiescent stage, low-grade neuroinflammation is associated with brain micro-lesions, secondarily implicated in a feeling of persistent fatigue [6]. The causal link between inflammation and fatigue in inactive IBD seems inconsistent and suffers from confounding variables [7,8]. Another view suggests that iron and micronutrient deficits are prevalent in people with IBD and may lead to anemia, contributing to fatigue [6]. Also, the microbiota and metabolomic profile of fatigued patients with IBD suggest a significant role for microbiota–gut–brain axis impairments in fatigue experience [9]. Fatigue can also be perceived because of cost-benefit miscalculations involving reward-based decision-making [10]. This is assessed at a cognitive level to integrate identified goal values leading to action [10]. Manifestations of impaired reward decision-making networks are classified as motivational and hedonic disorders [11] and are part of the negative syndrome in psychiatric semiology. They comprise avolition (a lack of motivation/ability to initiate or continue a goal-directed activity) and anhedonia (inability to experience pleasure and/or a reduced interest in things that used to give pleasure). These symptoms belong to the negative symptoms that are highly prevalent in schizophrenia, reported in 60% of patients [11], and in depressive disorder, with 81% of a first-episode population [12]. These symptoms are linked to the dysregulation of the nucleus accumbens—an essential structure involved in reward and motivation pathways [13]. They are linked to the alteration of brain structures involved in motivation belonging to the reward circuit, mainly constituting dopaminergic pathways [14]. Sub-symptoms of avolition and anhedonia are reported in the first episode of schizophrenia with respective prevalences of 53% and 16%, and in the first episode of major depressive disorder with respective prevalences of 50% and 32% [12]. To explain the persistence of fatigue experience in quiescent IBD, we hypothesize the existence of mental features, such as those found in psychiatric syndromes, that will engage to think of fatigue in a transdisciplinary way. Our wish is to identify targets that should be addressed for innovative treatment strategies.

## 2. Materials and Methods

### 2.1. Study Population

Patients were consecutively recruited from the University Hospital of Saint Etienne (France) in the Gastroenterology Department from the outpatient clinic between February 2021 and March 2022. Patients were all over 18 and diagnosed with IBD (CD or UC). The Crohn’s disease Activity Index (CDAI) [15] was use to quantify the symptoms of patients. It evaluates the number of liquid stool each day for 7 days, abdominal pain rated from 0 to 3 each day for 7 days, the general wellbeing rated from 0 to 4 each day for 7 days, the presence of complication: joint pain, uveitis, cutaneus localization, anal fissures or fistulae, fever during the previous week, the consumption of medication for diarrhea, the presence of an abdominal pass, the hematocrit results, and the deviation from the standard weight. The Mayo score [16] was use to assess the symptoms of ulcerative colitis. This scale evaluates the stool frequency, the rectal bleeding characteristic, the mucosal appearance at endoscopy, and a rating of disease activity by the physician. Quiescent IBD is characterized by a CDAI score ≤ 150 for patients with CD, and a Mayo score ≤ 2 for patients with UC; a CRP level below 5 mg/L of blood and calprotectin concentration over the last 6 month below 250 µg/g of stool is recommended in France (https://www.getaid.org/). Exclusion criteria consider patients presenting biological anemia, other current diagnoses of inflammatory diseases or major depressive disorder, or a diagnosis of fibromyalgia and chronic fatigue syndrome. Patients with ongoing depressive symptomatology (identified by a HADS score above 11), which may represent a case of undiagnosed major depressive disorder, or symptoms not reported by the patients, were interviewed by a psychiatrist to check whether they presented symptomatology compatible with a diagnosis of major depressive disorder and should therefore be excluded from the analysis. Groups were constituted following answers to five questions about the experience of fatigue at the time of recruitment by the patient, considering one group. Results were used to categorize patients into a group without fatigue (-NF) and another group presenting fatigue (-F). The study is explorative and consists of a unique evaluation using self-administered questionnaires. Patients’ clinical, biological, and sociodemographic characteristics were obtained from the medical file of the gastro-enterology department.

### 2.2. Assessment

A self-administered questionnaire was proposed online using RedCap software, version 10 [17]. Missing data can be a concern when evaluating a score of dimensions. Missing data were ignored in the score calculation when allowed by the questionnaire method. When more than 40% of the data were missing for all questionnaires, the subject was excluded from the analysis. Chalder’s fatigue questionnaire (CFQ) measured the experience of fatigue. A total of 11 items are divided into two dimensions: physical fatigue (7 items) and mental fatigue (4 items). Response choice consists of four propositions: (0 = better than usual, 1 = no more than usual, 2 = more than usual, 3 = worse than usual). Scoring could be conducted using two methods: to assess fatigue cases and not fatigue cases, the score is counted as 0 and 1 corresponds to 0 points, and 2 and 3 corresponds to 1 point. Fatigue cases are considered for a score of 4 or more. The Likert scale of scoring leads to a gradation and evaluation of the severity of fatigue experienced: the level of fatigue is correlated with higher scores [18]. The IBD-Disk was chosen to assess the disability associated with IBD’s symptoms by 10 items corresponding to the dimensions in which IBD causes difficulties in daily life (abdominal pain, regulating defecation, interpersonal interactions, work and study, sleep, energy, emotions, body image, sexual functions, joint pain). Answer propositions follow the Likert scale with 10 points from “absolutely not agree” to “totally agree”. The IBD-Disk score is then calculated in addition to all rating items. Higher scores correspond to higher disability in the dimension. An overall IBD-Disk score equal to or superior to 40 is considered for patients with moderate to severe disabilities [19]. The Hospital Anxiety and Depression Scale (HADS) was used to screen symptoms of anxiety and/or depression syndrome. Seven items are dedicated to each anxiety and depression syndrome subscales. Scoring consists of a Likert scale rating from 0 to 3, with some reverse scores for the chosen items. In the original publication, a score of 0 to 7 for either subscale was regarded as in the normal range, a score of 11 or higher indicating probable presence (‘caseness’) of a mood disorder, and a score of 8 to 10 being suggestive of the presence of the state syndrome [20]. As reported by Pais-Ribiero, the HADS manual indicates that a score between 0 and 7 is “normal”, between 8 and 10 “mild”, between 11 and 14 “moderate”, and between 15 and 21 “severe”. [21]. We only consider a score of 11 and over as a specific anxiety or depressive disorder symptomatology for a compatible diagnosis of the related disorder. The quality of life experienced by the patient was assessed by the Short Form Health Survey (SF-36), presenting 36 items evaluating eight dimensions of quality of life related to health: physical functioning, role limitations due to physical health, role limitation due to emotional problems; energy/fatigue; emotional well-being; social functioning; pain; general health: the lower score, the more severe the disability. Answers follow a Likert scale with seven propositions from “absolutely not” to “absolutely”. Score measures take account of the weight of items and are assessed from 0 to 100 after calculation with the rules given by the authors [22]. Negative symptoms were measured using the Self-evaluation of Negative Symptoms (SNS) scale [23]. This scale evaluates five subdomains with 20 items corresponding to negative symptoms that characterize the negative syndromes of psychiatric syndrome: social withdrawal; diminished emotional range; alogia; avolition; anhedonia. It had been validated for assessing negative symptoms in schizophrenia suffering patients. The subdomains are considered by scoring four items using a Likert scale with a score from 0 to 2 corresponding to “0 = absolutely not agree, 1 = somewhat agree, 2 = strongly agree). Higher scores correspond to a more substantial presence of negative symptoms in each dimension and in total score [24]. All surveys and methodology of scoring are available in the Supplementary Material and Methods section.

### 2.3. Statistical Analysis

Descriptive analysis was performed to describe variables in terms of mean, median, standard deviation, variance, minimum and maximum variables, frequency, and normality. A univariate analysis was conducted for psychometric test scores between groups reporting fatigue (-F) and those not reporting fatigue (-NF) and within each diagnostic category (CD and UC). Means were compared regarding normality using the unpaired *t*-student test or Mann–Whitney U test. To test independence and association between variables, contingency table tests were performed using the χ^2^ test or Fisher exact test regarding normality. The significance of the results is defined by a *p* value ≤ 0.05. A multi-way ANOVA (MANOVA) test compared multivariate sample means, considering an alpha risk-adjusted to 1%. The statistical model of multiple logistic regression was used with the dependent variable “fatigue declaration”. The independent variables were chosen according to their contribution to the PCA components in extreme quartiles: <−0.75 and >0.75. The classification method used was the area under the ROC curve. Correlation assessments were conducted using a two-tailed *p*-value Spearman nonparametric test. Missing data rows were omitted (n = 0). Results are expressed using r and a 95% confidence interval. A simple logistic regression analysis was performed with the dependent variable “fatigue declaration” and the independent variable “SNS score Avolition”. Statistical analysis and graphical representation were carried out using GraphPad Prism10 (San Diego, CA, USA).

## 3. Results

### 3.1. Fatigue Experience Is Associated with Disability and Low Quality of Life

**Figure 1:**
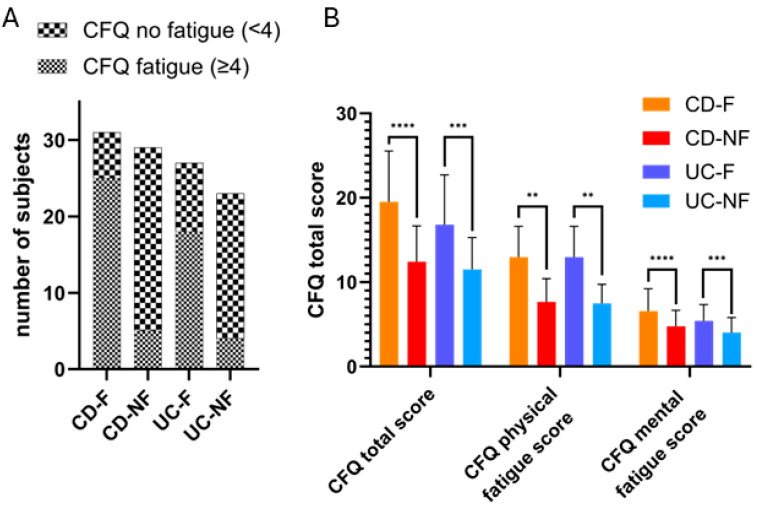
Patients were divided into two subgroups considering their declaration of fatigue experience. (**A**) Results of the Chalder’s fatigue questionnaire regarding the number of subjects reaching the score for fatigue objectivation by the scale (4). (**B**) Comparison of Chalder’s fatigue questionnaire mean score and mean sub-scale results regarding cohort categories. (CFQ: Chalder’s fatigue questionnaire; CD-NF: Crohn’s disease—not fatigued; CD-F: Crohn’s disease—fatigued; UC-NF: ulcerative colitis—not fatigued; UC-F: ulcerative colitis—fatigued; **: *p* < 0.01, ***: *p* = 0.0001, ****: *p* < 0.0001).

A total of 110 patients were recruited for the study. Among them, 60 were diagnosed with Crohn’s disease (CD), with 51% (n = 31) reporting fatigue (CD-F), and 49% (n = 29) not reporting fatigue (CD-NF). Additionally, 50 patients were diagnosed with ulcerative colitis (UC), of whom 54% (n = 27) declared experiencing fatigue (UC-F), and 46% (n = 23) did not (UC-NF). The groups were defined based on self-reported fatigue, which was correlated with our various metrics concerning fatigue (CFQ = Chalder’s fatigue questionnaire) (Figure 1). Clinical data of the population are described in Table 1.

**Figure 2:**
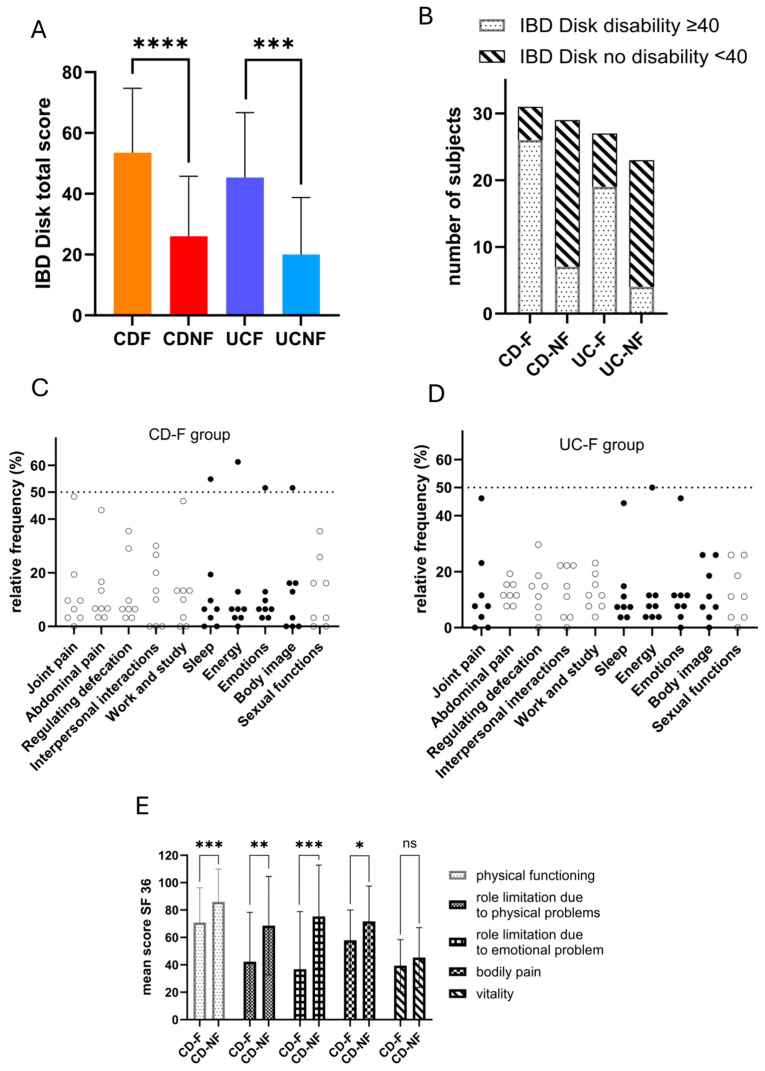
(**A**) Graphic presentation of IBD-Disk total score according to fatigue experience groups in each disease. (**B**) Graphic presentation of several subjects presenting an IBD-Disk total score representing a disability (≥40) in groups constituted following the declaration of fatigue experience. In total, 83.9% (n = 26) of patients in the CD-F group and 65.5% (n = 19) of patients in the UC-F group present moderate to severe disability. (**C**) Graphic representation of the relative frequency of IBD-Disk score following disability dimensions for the CD-F group. (**D**) Graphic representation of the relative frequency of IBD-Disk score following disability dimensions for the UC-F group. (**E**) Graphic presentation of SF-36 mean score in CD cohort regarding fatigue experience declaration for selected dimensions. (SF-36: 36 Short Form Health Survey, IBD: inflammatory bowel disease; CD-NF: Crohn’s disease—not fatigued; CD-F: Crohn’s disease—fatigued; UC-NF: ulcerative colitis—not fatigued; UC-F: ulcerative colitis—fatigued, *: *p* < 0.05; **: *p* < 0.01; ***: *p* ≤ 0.001; ****: *p* < 0.0001). ns: no significant.

As expected, disability assessed using IBD-Disk is highly consistent with fatigue declarations (Figure 2A). Disability and CFQ total scores (≥4) significantly associated in both the CD group (*p* < 0.0001) and UC group (*p* = 0.0002) (Figure 2B). According to the IBD-Disk assessment, dimensions of disability achieving a rating ≥7 are considered to contribute the most to overall disability. In the CD-F group, the energy dimension presents the highest relative frequency of score ≥7 (61.3%), followed by the sleep dimension (54.8%), and then emotion and body image dimensions (51.6% each) (Figure 2C). Similarly, in the UC-F group, the energy dimension had the highest relative frequency of score ≥ 7 (50%), followed by joint pain and emotion dimensions (46.2% each) and then the sleep dimension (44.4%) (Figure 2D). Considering these results, fatigue’s principal components appear to be associated with mental health dimensions and disabilities. Significative differences concerning the quality of life of patients were only identified in the CD group affecting physical functioning, role limitations due to physical and emotional problems, and bodily pain (Figure 2E). No dimensions presented significant differences between UC-F and UC-NF groups. The absence of statistically significant differences between fatigue groups for the general health dimension (Supplementary Figure S1) allows us to conclude that in our sample, IBD patients experiencing fatigue do not necessarily perceive themselves as unhealthy. SF-36 results suggest that fatigue primarily affects quality of life by exacerbating physical disability in those experiencing fatigue.

### 3.2. Fatigue Is Associated with Psychiatric Symptoms: Anxiety, Avolition, and Anhedonia, but Not with Depression

**Figure 3:**
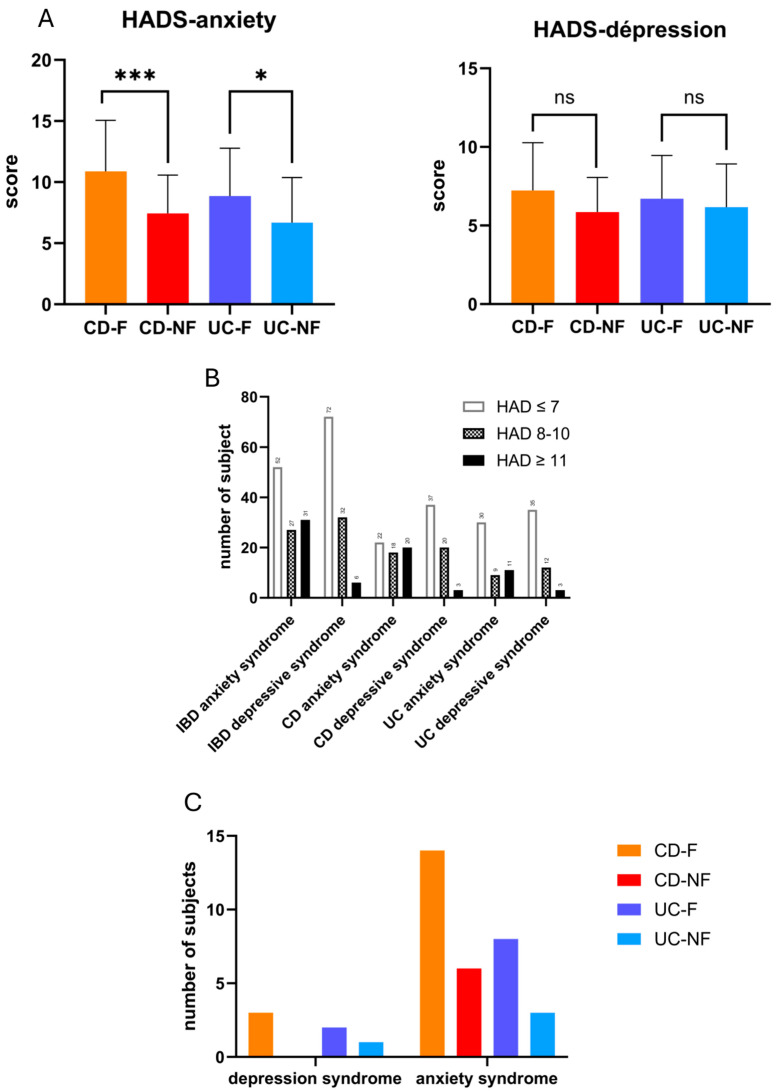
(**A**) Graphic presentation of hospital anxiety and depression scale anxiety score for groups declaring fatigue experience. (**B**) Graphic presentation of several subjects in each HADS score category regarding cohort groups of diseases. (**C**) Graphic presentation of several subjects regarding depression syndrome and anxiety syndrome in each group of the cohort. (HAD-D: Hospital Anxiety and Depression scale—Depression; Hospital Anxiety and Depression Scale—Anxiety; CD-NF: Crohn’s disease—not fatigued; CD-F: Crohn’s disease—fatigued; UC-NF: ulcerative colitis—not fatigued, ns: no significant; *: *p* < 0.05; ***: *p* = 0.0001).

To minimize confounding factors, we collected information about the previous history of depressive disorder. It concerns 24.5% of the cohort (CD-F: 32.2%; CD-NF: 24.1%; UC-F: 29.6%; UC-NF: 8.7%), and no significative frequency difference between groups (CD vs. UC *p* = 0.3766; CD-F vs. CD-NF *p* = 0.5725; UC-F vs. UC-NF *p* = 0.0850) appeared (Supplementary Table S1). We used the HAD scale to evaluate anxiety and depression symptoms. Only anxiety mean scores were revealed to be significantly higher in fatigue groups (CD-F: 10.87 (SD = 4.19), CD-NF: 7.45 (SD = 3.12), *p* = 0.0007; UC-F: 8.85 (SD = 3.92), UC-NF: 6.67 (SD = 3.67), *p* = 0.03) (Figure 3A). Symptoms intensity cut-off (> 11) allowed us to categorize patients in caseness. In the IBD population, depressive symptoms were reported as absent in 72 subjects (65.5%), uncertain in 32 subjects (29.1%), and probably present in 6 subjects (5.5%) (Figure 3B). After a clinical psychiatric evaluation, none of those six patients received the diagnosis of depressive disorder according to DSM5 criteria [25] and matched to exclusion criteria. The prevalence of symptoms in the group regarding the declaration of fatigue experience concluded with unexpected results. Regarding depressive symptoms probably present, only three patients (9.6%) in the CD-F group (mean score of 13.6/21) and two patients (7.1%) in the UC-F group (mean score of 12/21) could be classified as caseness. Regarding anxiety symptoms, 14 patients (45%) from the CD-F group (mean score = 14.7/21) and 8 patients (30%) from the UC-F group (mean score: 13.75/21) could be classified as caseness (Figure 3C). Fisher’s exact test performed in the CD group concludes with the independence of fatigue and depressive symptoms scores (*p* = 0.2) and anxiety symptoms scores (*p* = 0.06), which present a tendency to note. Contrary to expectations, Fisher’s exact test indicated no significant relationship between fatigue and depression in either the CD (*p* = 0.2) or UC groups (*p* > 0.9). These findings challenge the assumption of a strong correlation between fatigue and depression.

**Figure 4:**
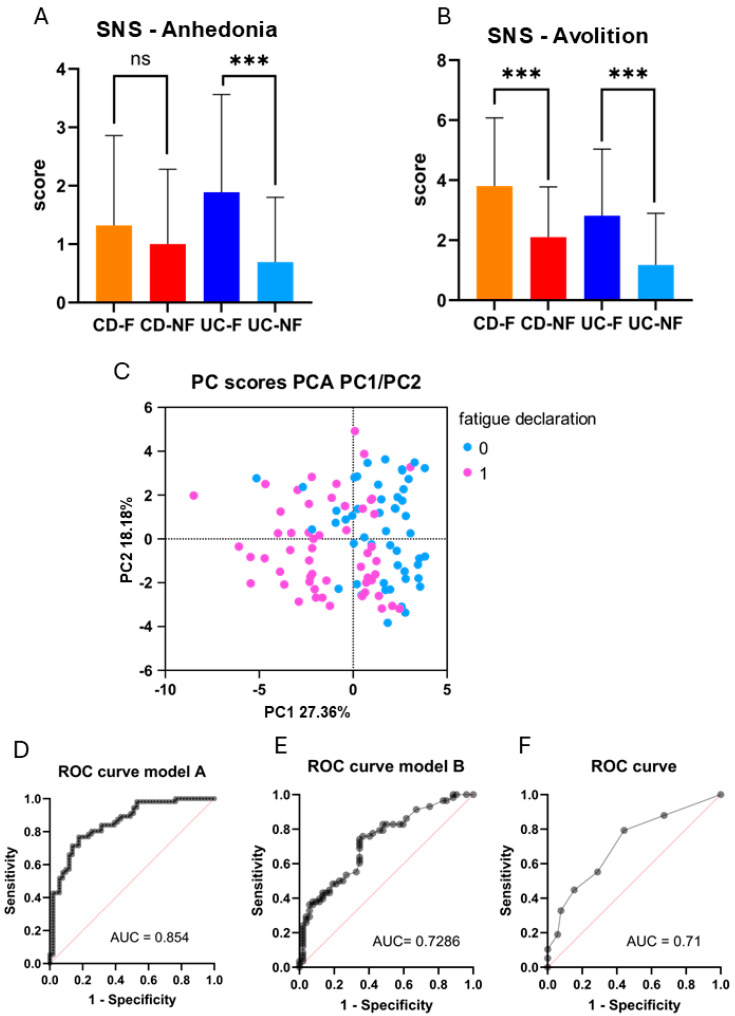
(**A**) Comparison of SNS score for anhedonia dimension between groups regarding fatigue declaration. (**B**) Comparison of SNS score for avolition dimension between groups regarding fatigue declaration. (**C**) Graphic presentation of principal component analysis results. (**D**) ROC curve expressed regarding multiple logistic regression model A. (**E**) ROC curve expressed regarding multiple logistic regression model B. (**F**) The ROC curve is expressed regarding the simple logistic regression model using the “SNS Avolition” score variate. (SNS: Self-evaluation of Negative Symptoms; CD-NF: Crohn’s disease—not fatigued; CD-F: Crohn’s disease—fatigued; UC-NF: ulcerative colitis—not fatigued; UC-F: ulcerative colitis—fatigued; ns: no significant; ***: *p* < 0.001).

Symptoms associated with motivation alteration were evaluated using the Self-evaluation of Negative Symptom (SNS) questionnaire. Regarding anhedonia (loss of ability to feel pleasure in activities), only UC patients presented a significant difference in the score (UC-F = 1.889 (SD = 1.672); UC-NF = 0.696 (SD = 1.105); *p* = 0.0051) (Figure 4A). About avolition (lack of motivation to initiate or persist in an activity), scores differed significantly between groups of the two IBD conditions: (CD-F = 3.806 (SD = 2.272); CD-NF = 2.103 (SD = 1.676), *p* = 0.0032) and (UC-F = 2.815 (SD = 2.219); UC-NF = 1.174 (SD = 1.723), *p* = 0.0029) (Figure 4B). We performed an analysis within subgroups of subjects considering their self-reported history of depressive disorder. These results remained unchanged when accounting for the history of depression (Supplementary Table S2). Analysis of contingencies revealed no significant association between depression and avolition in both the CD-F group (*p* = 0.5350) and the UC-F group (*p* = 0.1880). Avolition scores and CFQ total score were recorded in both the CD-F group (Pearson r = 0.5109; CI 95% [0.1912; 0.7326], *p* = 0.0033), and the UC-F group (Pearson r = 0.4254; CI 95% [0.0541; 0.6933]; *p* = 0.027). These findings indicate a moderate positive correlation between avolition and fatigue scores in the two groups declaring to experience fatigue. To state the association between avolition and fatigue, we conducted a multivariate analysis to investigate variables that significantly participate in fatigue declaration (details of variables in Table 1). The PCA analyses identified three major components (Figure 4C), and variables contributing to these dimensions (extreme quartiles) were included in multiple logistic regression models with “fatigue declaration” as the dependent variable (detailed in the Supplementary Materials and Methods). An initial multiple logistic regression model (Model A) revealed a strong association between self-reported fatigue experience and several variables of interest, including avolition, and yielded an area under the ROC curve of 0.8540, CI95% [0.7830; 0.9250] (Figure 4D). A simpler model using only “SNS Avolition” and “HADS Anxiety” scores achieved an AUC of 0.7286 (CI95% [0.6353; 0.8220]) (Figure 4E). A simple logistic regression analysis involved “fatigue declaration” as the dependent variable and “SNS score Avolition” as the independent variable. The model fits well, showing an area under ROC curve of 0.71, SD = 0.05 (Figure 4F). This model allows us to conclude that for a one-unit increase in avolition score, the risk of reporting fatigue experience increased by a multiplicative factor of 1.5 (calculate odds ratio β1 of 1.5 with IC95 = [1.2;1.9]). Both areas under ROC curves are close, even when isolating “SNS score Avolition”. Furthermore, correlation analysis by Spearman’s test concludes that the anxiety dimension in the IBD-Disk survey (r = 0.49, CI95% [0.32; 0.62]) and HADS survey (r = 0.43, CI95% [0.26; 0.58]) correlate with CFQ fatigue total score (Supplementary Figure S2). Overall, these results highlight a robust association between fatigue and avolition.

## 4. Discussion

To help understand and address fatigue and its burden, our study investigated the psychometric scales in patients with IBD. Based on our results, the fatigue experienced by patients in a quiescent state of IBD is characterized by avolition without the presence of a current depression disorder. The avolition score is significantly different between groups regarding reported fatigue experience. Moreover, fatigue is substantially associated with avolition, a relationship that appears to be independent of depression scores and a history of depressive disorder. Mental health issues are a common feature for patients with IBD, with meta-analysis [26] reporting a prevalence of 33.2% of anxiety symptoms and 21.6% of depression symptoms in patients with IBD. Regarding patients with quiescent IBD, 34.1% present anxiety syndromes and 18.2% show depression syndromes [27]. In our study, the prevalence of probable depressive syndrome (score ≥ 11) is 5.5% and 28.8% for anxiety syndrome (score ≥ 11), which is consistent with rates evaluated using HADS in a study by Stroie et al. in 2023 [4]. Recent studies attempting to treat fatigue in quiescent IBD with 5-hydroxytryptophan supplementation (serotonin precursor) have failed to show superiority over placebo, reinforcing the idea that this mechanism may be independent of depression disorder [28]. Unfortunately, our protocol did not collect information on antidepressant use or the type of biological treatments administered at the time of the survey, which may influence levels of fatigue experienced in a remission state [29]. It is worth noting that a tendency of dependence exists between patients from the CD-F group and anxiety syndrome, as unveiled by HADS.

Our assertion is entirely new and opens the way to pathophysiological hypotheses. Even in remitted IBD, we believe that the brain structures involved in motivational domains may be altered, contributing to the experience of fatigue associated with a lack of motivation. Interestingly and matching with our findings, a qualitative study in patients with CD (n = 35, 74% of subjects with fatigue experience) reported that fatigue was characterized by sensations consistent with “lack of energy”, “lack of stamina”, “sleepiness”, and mental fatigue, described as “difficulties concentrating/focusing”, “feeling depressed”, “lack of motivation”, and “zoning out” [30]. These symptoms named by patients as “fatigue” are often related to the motivational dimension of action and play a crucial role in the functional outcomes of psychiatric disorders. The experience of fatigue is partly conditioned by the brain system for motivation and reward functions. These symptoms may reflect disruptions in cognitive pathways and brain structures, particularly those involved in motivation (dopaminergic pathways in the reward system belonging to the positive valence domain).

Our work presents some limitations regarding the scales used. Fatigue was assessed solely with the CFQ, which is recommended in France by the HAS (Health High Authority), rather than the FACIT-F or MFI questionnaires commonly employed in other studies. Future protocols should consider incorporating the two scales to comprehensively evaluate fatigue. The SNS scale has not yet been validated in the population of patients with IBD. Despite these limitations, this approach introduces an innovative approach in the field, and we strongly believe that assessing the positive valence construct, which is relevant to schizophrenia, can be extended to other conditions within the framework of the research domains criteria approach for exploring brain functioning [31]. Studies in the field of addictology are discovering a potential for intestinal microbiota dysbiosis to disrupt the reward system [32]: key genera from the intestinal microbiota are associated with dopamine [33], and may even influence the regulation of the human social decision-making process [34]. The hypothesis of an intestinal microbiota specific to quiescent IBD patients suffering from fatigue has been validated [9], paving the way for an altered gut–brain axis function secondary to IBD flares, as suggested by Fairbrass et al. 2022 [35]. This establishes a link between the potential alteration of the central nervous system, manifested in cognitive and behavioral disorders in impaired goal-directed motivation (mediated by dopaminergic signaling in the reward system), and the immunopathology of IBD. Specific bacterial metabolites from strains known to modulate receptors, transporters, and targets of dopaminergic pathways, notably, *Prevotella*, *Bacteroides*, *Lactobacillus*, *Bifidobacterium*, *Clostridium*, *Enterococcus*, and *Ruminococcus* [33] have been found altered in abundance in the intestinal microbiota of patients with IBD [36]. Fortunately, some care strategies currently exist to improve fatigue experience and quality of life, including physical activity programs [37] and psychotherapeutic interventions [38,39]. So far, the treatment of negative symptoms has mostly been developed in the psychiatric field for schizophrenia, through the use of antipsychotics, antidepressants, and noninvasive brain stimulation [11,40].

## 5. Conclusions

This study provides for the first time elements about a strong association between avolition, a psychiatric symptom known to be related to the reward circuit of the brain, and the experience of fatigue in patients with a quiescent state of IBD. This finding is also independent of depressive symptoms that was not reported significantly in our cohort of patients. The transdiagnostic nature of avolition encourages further research into pathophysiology to identify precise targets that can serve as motivational stimulants and alleviate feelings of fatigue. Results from our study represent a new step toward innovative treatment targets for many patients experiencing fatigue or suffering from negative symptoms.

## Figures and Tables

**Table 1:** A: Sociodemographic and clinical characteristics for the CD-NF and CD-F cohort. (CD-NF: Crohn’s disease—not fatigued; CD-F: Crohn’s disease—fatigued; IBD: inflammatory bowel disease; SD: standard deviation; Q1: first quartile; Q3: third quartile; CRP: c-reactive protein; CDAI: Crohn’s disease Activity Index). ^1^: noninflammatory state is defined by CRP level below 5 mg/L of blood, and calprotectin concentration below 250 µg/g of stool. ^2^: Active IBD is defined by a CDAI score over 150, and a CRP level over 5 mg/L of blood, and calprotectin concentration over 250 µg/g of stool.

	Declaration of Fatigue	Sex Gender			Age					Family History of Depressive Disorder			Family History of IBD		Personal History of Depressive Disorder		Active Smoking		Cannabis Usage		Age of IBD Onset		
		male		female	18–29	30–39	40–49	50–59	> 60	no		yes	no	yes	no	yes	no	yes	no	yes	<16 (A1)	17–40 (A2)	>4 (A3)
CD	31	22		38	13	13	16	10	8	43		17	48	12	43	17	45	15	60	0	6	50	4
%	51.0	36.7		63.3	21.7	21.7	26.7	16.7	13.3	71.1		28.3	80	20	71.1	28.3	75	25	100	0	10	83.3	6.7
CD-NF		13		16	8	5	7	5	4	23		6	23	6	22	7	23	6	29	0	5	21	3
%		44.8		55.1	27.6	17.2	24.1	17.2	13.8	79.3		20.7	79.3	20.7	75.9	24.1	79.3	20.7	100	0	10.3	72.4	17.2
CD-F		9		22	5	8	9	5	4	20		11	25	6	21	10	22	9	31	0	1	29	1
%		29		70	16.1	25.8	29.0	16.1	12.9	64.5		35.5	64.5	35.5	67.7	32.3	67.7	32.3	100	0	3.23	93.5	3.2
UC	27	31		19	7	10	14	14	5	12		38	43	7	40	10	43	7	49	1	3	41	6
%	54.0	62		38	14	20	28	28	10	24		76	86	14	80	20	86	14	98	2	6	82	12
UC-NF		19		4	5	3	5	7	3	20		3	20	3	21	2	20	3	23	0	3	15	5
%		82.6		17.4	21.7	13.0	21.7	30.4	13.0	87.0		13.0	87.0	13.04	91.3	8.7	87.0	13.0	100	0	13.0	65.2	21.7
UC-F		12		15	2	7	9	7	2	18		9	23	4	19	8	23	4	26	1	0	26	1
%		44.4		55.6	7.41	25.9	33.3	25.9	7.4	66.7		33.3	85.2	14.8	70.4	29.6	85.2	14.8	96.3	3.7	0	96.3	3.7
	Number of years with IBD				Active IBD ^2^		Biological inflammatory marker dosage (CRP/calprotectin) ^1^			Actual treatment				Number of treatment failure					Surgery treatment history				
	mean (SD)	median [Q1;Q3]		interval	no	yes	No inflammation		inflammation	corticoids	Biologics	probiotics	without treatment		<2	3–4	>5		0		1–2	3–4	>5
CD	12.6 (9.75)	9.5 [5, 20]		0–40	60	0	59		1	0	59	0	1		36	12	12		38		17	0	5
%					100	0	98.3		1.67	0	98.3	0	1.67		60	20	20		63.3		28.3	0	8.3
CD-NF	11.83 (8.41)	9 [5.5; 14.5]		2–36	29	0	28		1	0	29	0	0		18	6	5		20		6	0	3
%					100	0	3.5		96.5		100				62.1	20.7	17.2		69		20.7	0	10.3
CD-F	13.35 (10.95)	10 [4; 23]		0–40	31	0	31		0	0	30	0	1		18	6	7		18		11	0	2
%					100	0	100		0	0	96.77	0	3.23		58.1	19.3	22.6		58.1		35.5	0	6.45
UC	11,1 (7.26)	10.5 [6, 15]		1–32	0	50	48		2	0	46	2	1		25	20	2		45		3	2	0
%					0	100	96		4	0	92	4	2		50	40	10		90		6	4	0
UC-NF	9.91 (6.65)	8 [4; 15]		1–23	0	23	22		1	0	20	1	1		13	9	1		21		1	1	0
%					0	100	95.65		4.35	0	90.91	4.55	4.55		56.5	39.1	4.3		91.3		4.3	4.3	0
UC-F	12.11 (7.68)	11 [7; 15]		1–32	0	27	26		1	0	26	1	0		12	11	4		24		2	1	0
%					0	100	96.3		3.7	0	96.3	3.7	0		44.4	40.7	14.8		88.9		7.4	3.7	0
				Age of IBD onset			Montréal score-localization								Montréal score-behavior								
	CDAI score			A1	A2	A3	L1		L2	L3	L4				B1				B2		B3		
	<150	150–220	>220	<16	17–40	>40	small intestine		colon	ileocolitis	upper digestive tract				Non-stricturing				stricturing		penetrating		
CD	59	1	0	6	50	4	21		11	28	0				43				11		6		
%	98.3	1.67	0	10	83.3	6.67	35		18.34	46.7	0				71.67				18.33		10		
CD-NF	29	0	0	5	21	3	13		5	11	0				19				5		5		
%	100	0	0	10.3	72.4	17.2	44.8		17.2	38					65.5				17.25		17.25		
CD-F	30	1	0	1	29	1	8		6	17	0				22				6		1		
%	96.67	3.33	0	3.23	93.55	3.23	25.81		19.35	54.84	0				75.86				20.69		3.45		
						Age of IBD onset				Mayo interpretation					Montréal score-extent								
		Mayo score				A1	A2		A3						E1				E2		E3		
		0	1	2		<16	17–45		>45	inactive UC					procolitis				left colitis		pancolitis		
UC		47	1	2		3	41		6						7				21		20		
%		94	2	4		6	82		12						14.59				43.74		41.67		
UC-NF		22	0	1		3	15		5	23					2				10		10		
%		95.65	0	4.35		13.04	65.22		21.74	100					9.09				45.45		45.45		
UC-F		25	1	1		0	26		1	27					5				11		10		
%		92.59	3.7	3.7		0	96.3		3.7	100					19.23				42.31		38.46		

## Data Availability

Raw data are available over request to the corresponding author.

## Supplementary Materials

The following supporting information can be downloaded at: https://www.mdpi.com/article/10.3390/biomedicines13010125/s1, Figure S1: Graphic representation of SF-36 for general health dimension; Figure S2: Pearson correlation table from PCA; Table S1: Description of cohort characteristics regarding previous history of depressive disorder; Table S2: Description of variables involved in the multivariable statistical analysis. References [41,42,43,44,45,46,47,48] are cited in the supplementary materials.
