# Supplementary material for: Avolition Characterizes the Chronic Fatigue Experienced in Quiescent Inflammatory Bowel Disease
Source: Biomedicines. 2025 Jan 7;13(1):125. doi: 10.3390/biomedicines13010125 (PMC11761293; doi:10.3390/biomedicines13010125)
Supplement: Supplementary file 1 [file biomedicines-13-00125-s001.zip › supplementary material and methods.pdf]

Supplementary Material and Methods

STROBE Statement—checklist of items that should be included in reports of observational studies

	Item No	Recommendation	Article check
**Title and abstract**	1	(*a*) Indicate the study's design with a commonly used term in the title or the abstract	✓
		(*b*) Provide in the abstract an informative and balanced summary of what was done and what was found	✓
**Introduction**			
Background/rationale	2	Explain the scientific background and rationale for the investigation being reported	✓
Objectives	3	State specific objectives, including any prespecified hypotheses	✓
**Methods**			
Study design	4	Present key elements of study design early in the paper	✓
Setting	5	Describe the setting, locations, and relevant dates, including periods of recruitment, exposure, follow-up, and data collection	✓
Participants	6	(*a*) *Cohort study*—Give the eligibility criteria, and the sources and methods of selection of participants. Describe methods of follow-up<br>*Case-control study*—Give the eligibility criteria, and the sources and methods of case ascertainment and control selection. Give the rationale for the choice of cases and controls<br>*Cross-sectional study*—Give the eligibility criteria, and the sources and methods of selection of participants	✓
		(*b*) *Cohort study*—For matched studies, give matching criteria and number of exposed and unexposed<br>*Case-control study*—For matched studies, give matching criteria and the number of controls per case	NA
Variables	7	Clearly define all outcomes, exposures, predictors, potential confounders, and effect modifiers. Give diagnostic criteria, if applicable	✓
Data sources/ measurement	8*	For each variable of interest, give sources of data and details of methods of assessment (measurement). Describe comparability of assessment methods if there is more than one group	✓

Bias	9	Describe any efforts to address potential sources of bias	✓
Study size	10	Explain how the study size was arrived at	✓
Quantitative variables	11	Explain how quantitative variables were handled in the analyses. If applicable, describe which groupings were chosen and why	✓
Statistical methods	12	(*a*) Describe all statistical methods, including those used to control for confounding	✓
		(*b*) Describe any methods used to examine subgroups and interactions	✓
		(*c*) Explain how missing data were addressed	✓
		(*d*) *Cohort study*—If applicable, explain how loss to follow-up was addressed<br>*Case-control study*—If applicable, explain how matching of cases and controls was addressed<br>*Cross-sectional study*—If applicable, describe analytical methods taking account of sampling strategy	NA
		(*e*) Describe any sensitivity analyses	NA
**Results**			
Participants	13*	(a) Report numbers of individuals at each stage of study—eg numbers potentially eligible, examined for eligibility, confirmed eligible, included in the study, completing follow-up, and analysed	✓
		(b) Give reasons for non-participation at each stage	NA
		(c) Consider use of a flow diagram	NA
Descriptive data	14*	(a) Give characteristics of study participants (eg demographic, clinical, social) and information on exposures and potential confounders	✓
		(b) Indicate number of participants with missing data for each variable of interest	✓
		(c) *Cohort study*—Summarise follow-up time (eg, average and total amount)	NA
Outcome data	15*	*Cohort study*—Report numbers of outcome events or summary measures over time	NA
		Case-control study—Report numbers in each exposure category, or summary measures of exposure	NA
		Cross-sectional study—Report numbers of outcome events or summary measures	NA
Main results	16	(*a*) Give unadjusted estimates and, if applicable, confounder-adjusted estimates and their precision (eg, 95% confidence interval). Make clear which	✓

		confounders were adjusted for and why they were included	
		(*b*) Report category boundaries when continuous variables were categorized	NA
		(*c*) If relevant, consider translating estimates of relative risk into absolute risk for a meaningful time period	NA
Other analyses	17	Report other analyses done—eg analyses of subgroups and interactions, and sensitivity analyses	✓
**Discussion**			
Key results	18	Summarise key results with reference to study objectives	✓
Limitations	19	Discuss limitations of the study, taking into account sources of potential bias or imprecision. Discuss both direction and magnitude of any potential bias	✓
Interpretation	20	Give a cautious overall interpretation of results considering objectives, limitations, multiplicity of analyses, results from similar studies, and other relevant evidence	✓
Generalisability	21	Discuss the generalisability (external validity) of the study results	✓
**Other information**			
Funding	22	Give the source of funding and the role of the funders for the present study and, if applicable, for the original study on which the present article is based	✓

Questionnaires :

Chalder's fatigue questionnaire

The Chalder's Fatigue Questionnaire(1) (CFQ) measured the experience of fatigue and its characteristics. 11 items are divided into two dimensions: physical fatigue (7 items) and mental fatigue (4 items). Response choice consists of 4 propositions: (0= = better than usual, 1 = no more than usual, 2 = more than usual, 3 = worse than usual). Scoring could be done using two methods: to assess fatigue cases and not fatigue cases, the score is counted as 0 and 1 corresponds to 0 points, and 2 and 3 corresponds to 1 point. Fatigue cases are considered for a score of 4 or more(2). The Lickert scale of scoring leads to a gradation and evaluation of the severity of fatigue experienced: the level of fatigue is correlated with higher scores. Discriminative properties were determined with an area under the curve for ROC of 0.91(3). Missing data were ignored in the calculation of the fatigue score.

chalder fatigue scale

name: ______________________________ date: _______

We would like to know more about any problems you have had with feeling tired, weak or lacking in energy in the last month. Please answer ALL the questions by ticking the answer which applies to you most closely. If you have been feeling tired for a long while, then compare yourself to how you felt when you were last well. Please tick only one box per line.

	less than usual	***no more than usual***	***more than usual***	***much more than usual***
do you have problems with tiredness?				
do you need to rest more?				
do you feel sleepy or drowsy?				
do you have problems starting things?				
do you lack energy?				
do you have less strength in your muscles?				
do you feel weak?				
do you have difficulties concentrating?				
do you make slips of the tongue when speaking?				
do you find it more difficult to find the right word?				
	better than usual	***no worse than usual***	***worse than usual***	***much worse than usual***
how is your memory?				

This scale can be scored "bimodally" with columns representing 0, 0, 1 & 1 and a range from 0 to 11 with a total of 4 or more qualifying for "caseness". Alternatively it can be scored in "Likert" style 0, 1, 2 & 3 with a range from 0 to 33. Mean "bimodal" score for CFS sufferers was 9.14 (SD 2.73) and for a community sample 3.27 (SD 3.21). Mean "Likert" score was 24.4 (SD 5.8) and 14.2 (SD 4.6).

total (0-33) =

Cella, M. and T. Chalder (2010). "Measuring fatigue in clinical and community settings." J Psychosom Res 69(1): 17-22. This study involved 361 CFS sufferers and 1615 individuals from the community. Average age was in the 30's. Fatigue levels were similar for males and females. A score of 29 discriminated between CFS sufferers and the community sample in 96% of cases and a score in the 30's discriminated in 100% of cases. The CFS sufferers also scored a mean of 26.99 on the Work & Social Adjustment Scale (W&SAS) with a SD of 8.6 (i.e. about 70% scoring between 18.4 and 35.6).

The IBD-Disk:

The experience of symptoms of the IBD was evaluated using the IBD-Disk(4). This questionnaire is the French version of the IBD Disability Index(5). This questionnaire evaluates the disability by 10 items corresponding to the dimensions in which IBD causes difficulties in daily life. Answer propositions follow the Lickert scale with 10 points from "absolutely not agree" to "totally agree". The IBD-Disk score is then calculated in addition to all rating items. Higher scores correspond to higher disability in the dimension. An overall IBD-Disk score equal to or superior to 40 is considered for patients with moderate to severe disabilities(6). Missing data were ignored for the calculation of the impact score.

For each of the ten statements below, score your level of agreement on a scale of 0 to 10.
Circle your scores on the coloured disc.

Absolutely disagree					Neither agree or disagree					Absolutely agree
0	1	2	3	4	5	6	7	8	9	10

In the last week, because of my Crohn's disease or ulcerative colitis...

**Abdominal pain**	...I have had aches or pains in my stomach or abdomen
**Regulating defecation**	...I have had difficulty coordinating and managing defecation, including choosing and getting to an appropriate place for defecation and cleaning myself afterwards
**Interpersonal interactions**	...I have had difficulty with personal relationships and/or difficulty participating in the community
**Education and work**	...I have had difficulty with school or studying activities, and/or difficulty with work or household activities
**Sleep**	...I have had difficulty sleeping, such as falling asleep, waking up frequently during the night or waking up too early in the morning
**Energy**	...I have not felt rested and refreshed during the day, and have felt tired and without energy
**Emotions**	...I have felt sad, low or depressed, and/or worried or anxious
**Body image**	...I have not liked the way my body or body parts look
**Sexual functions**	...I have had difficulty with the mental and/or physical aspects of sex
**Joint pain**	...I have had pains in the joints of my body

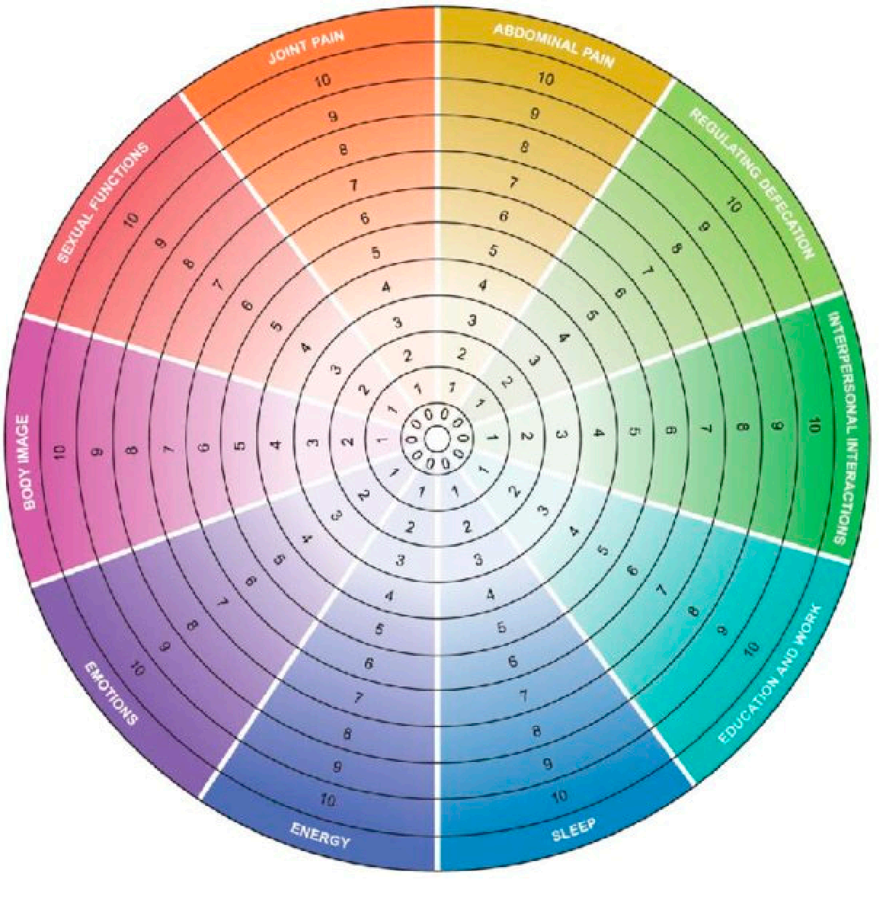

The Hospital Anxiety and Depression Scale (HADS)

The Hospital Anxiety and Depression Scale (HADS) scale is used to screen symptoms of anxiety and/or depression syndrome. We use this scale to evaluate the proportion of anxiety and depressive syndrome in our cohort of patients experiencing fatigue. Seven items are dedicated to each anxiety and depression syndrome subscales. Scoring consists of a Lickert scale rating from 0 to 3, with some reverse scores for the chosen items. In the original publication, a score of 0 to 7 for either subscale was regarded as in the normal range, a score of 11 or higher indicating probable presence ('caseness') of a mood disorder, and a score of 8 to 10 being suggestive of the presence of the state syndrome (7). As reported by Pais-Ribiero, the HADS manual indicates that a score between 0 and 7 is "normal," between 8 and 10 "mild," between 11 and 14 "moderate," and between 15 and 21 "severe."(8). We only consider a score of 11 and over as a specific anxiety or depressive disorder symptomatology for a compatible diagnosis of the related disorder. Patients with a score above 11 were secondly interviewed by a psychiatrist to establish a diagnosis of current depressive disorder, that is representing an exclusion criterion, and necessitating patient exclusion. According to recommendation from Bell et al. 2016, we decide to ignore missing data in calculation of subscales rating(9).

Hospital Anxiety and Depression Scale (HADS)

Tick the box beside the reply that is closest to how you have been feeling in the past week.
Don't take too long over you replies: your immediate is best.

D	A		D	A	
		**I feel tense or 'wound up':**			**I feel as if I am slowed down:**
	3	Most of the time	3		Nearly all the time
	2	A lot of the time	2		Very often
	1	From time to time, occasionally	1		Sometimes
	0	Not at all	0		Not at all
		**I still enjoy the things I used to enjoy:**			**I get a sort of frightened feeling like 'butterflies' in the stomach:**
0		Definitely as much		0	Not at all
1		Not quite so much		1	Occasionally
2		Only a little		2	Quite Often
3		Hardly at all		3	Very Often
		**I get a sort of frightened feeling as if something awful is about to happen:**			**I have lost interest in my appearance:**
	3	Very definitely and quite badly	3		Definitely
	2	Yes, but not too badly	2		I don't take as much care as I should
	1	A little, but it doesn't worry me	1		I may not take quite as much care
	0	Not at all	0		I take just as much care as ever
		**I can laugh and see the funny side of things:**			**I feel restless as I have to be on the move:**
0		As much as I always could		3	Very much indeed
1		Not quite so much now		2	Quite a lot
2		Definitely not so much now		1	Not very much
3		Not at all		0	Not at all
		**Worrying thoughts go through my mind:**			**I look forward with enjoyment to things:**
	3	A great deal of the time	0		As much as I ever did
	2	A lot of the time	1		Rather less than I used to
	1	From time to time, but not too often	2		Definitely less than I used to
	0	Only occasionally	3		Hardly at all
		**I feel cheerful:**			**I get sudden feelings of panic:**
3		Not at all		3	Very often indeed
2		Not often		2	Quite often
1		Sometimes		1	Not very often
0		Most of the time		0	Not at all
		**I can sit at ease and feel relaxed:**			**I can enjoy a good book or radio or TV program:**
	0	Definitely	0		Often
	1	Usually	1		Sometimes
	2	Not Often	2		Not often
	3	Not at all	3		Very seldom

Please check you have answered all the questions

Scoring:
Total score: Depression (D) ___________ Anxiety (A) _____________
0-7 = Normal
8-10 = Borderline abnormal (borderline case)
11-21 = Abnormal (case)

The Short Form Health Survey (SF-36)

Quality of life experienced by the patient was assessed by the Short Form Health Survey (SF-36)(10), presenting 36 items evaluating 8 dimensions of quality of life related to health: physical functioning, role limitations due to physical health, role limitation due to emotional problems; energy/fatigue; emotional well-being; social functioning; pain; general health: the lower score, the more disability. Answers follow a Lickert scale with seven propositions from “absolutely not” to “absolutely”. Score measures take account of the weight of items and evolve from 0 to 100 after calculation with the rules given by authors(11).

Medical Outcomes Study Questionnaire Short Form 36 Health Survey (SF-36)

About: The SF-36 is an indicator of overall health status.

Items: 10

Reliability: Most of these studies that examined the reliability of the SF_36 have exceeded 0.80 (McHorney et al., 1994; Ware et al., 1993). Estimates of reliability in the physical and mental sections are typically above 0.90.

Validity: The SF-36 is also well validated.

Scoring:

The SF-36 has eight scaled scores; the scores are weighted sums of the questions in each section. Scores range from 0 - 100

Lower scores = more disability, higher scores = less disability

Sections:

- Vitality
- Physical functioning
- Bodily pain
- General health perceptions
- Physical role functioning
- Emotional role functioning
- Social role functioning
- Mental health

References:

McHorney CA, Ware JE, Lu JFR, Sherbourne CD. The MOS 36-Item Short-Form Health Survey (SF-36®): III. tests of data quality, scaling assumptions and reliability across diverse patient groups. *Med Care*1994; 32(4):40-66.

Ware JE, Snow KK, Kosinski M, Gandek B. *SF-36® Health Survey Manual and Interpretation Guide*. Boston, MA: New England Medical Center, The HealthInstitute, 1993.

Ware JE, Sherbourne CD. The MOS 36-Item Short-Form Health Survey (SF-36®): I. conceptual framework and item selection. Med Care 1992; 30(6):473-83.

Medical Outcomes Study Questionnaire Short Form 36 Health Survey

This survey asks for your views about your health. This information will help keep track of how you feel and how well you are able to do your usual activities. Thank you for completing this survey! For each of the following questions, please circle the number that best describes your answer.

**1. In general, would you say your health is:**	
Excellent	1
Very good	2
Good	3
Fair	4
Poor	5
**2. Compared to one year ago,**	
Much better now than one year ago	1
Somewhat better now than one year ago	2
About the same	3
Somewhat worse now than one year ago	4
Much worse now than one year ago	5

3. The following items are about activities you might do during a typical day. Does your health now limit you in these activities? If so, how much?

(Circle One Number on Each Line)

	**Yes, Limited a Lot (1)**	**Yes, Limiteda Little (2)**	**No, Not limited at All (3)**
a. **Vigorous activities**, such as running, lifting heavy objects, participating in strenuous sports	1	2	3
b. **Moderate activities**, such as moving a table, pushing a vacuum cleaner, bowling, or playinggolf	1	2	3
c. Lifting or carrying groceries	1	2	3
d. Climbing **several** flights of stairs	1	2	3
e. Climbing **one** flight of stairs	1	2	3
f. Bending, kneeling, or stooping	1	2	3

g. Walking **more than a mile**	1	2	3
h. Walking **several blocks**	1	2	3
i. Walking **one block**	1	2	3
j. Bathing or dressing yourself	1	2	3

4. During the **past 4 weeks**, have you had any of the following problems with your work or other regular daily activities **as a result of your physical health**?

(Circle One Number on Each Line)

	**Yes (1)**	**No (2)**
a. Cut down the amount of time you spent on work or other activities	1	2
b. **Accomplished less** than you would like	1	2
c. Were limited in the **kind** of work or other activities	1	2
d. Had **difficulty** performing the work or other activities (for example, it took extra effort)	1	2

5. During the **past 4 weeks,** have you had any of the following problems with your work or other regular daily activities **as a result of any emotional problems** (such as feeling depressed or anxious)?

(Circle One Number on Each Line)

	**Yes**	**No**
a. Cut down the amount of time you spent on work or other activities	1	2
b. **Accomplished less** than you would like	1	2
c. Didn't do work or other activities as **carefully** as usual	1	2

**6. During the past 4 weeks, to what extent has your physical health or emotional problems interfered with your normal social activities with family, friends, neighbors, or groups?**	
Not at all	1
Slightly	2
Moderately	3
Quite a bit	4
Extremely	5

**7. How much bodily pain have you had during the past 4 weeks?**	
None	1
Very mild	2
Mild	3
Moderate	4
Severe	5
Very severe	6
**8. During the past 4 weeks, how much did pain interfere with your normal work (including both work outside the home and housework)?**	
Not at all	1
A little bit	2
Moderately	3
Quite a bit	4
Extremely	5

These questions are about how you feel and how things have been with you **during the past 4 weeks**. For each question, please give the one answer that comes closest to the way you have been feeling. **(Circle One Number on Each Line)**

9. How much of the time during the **past 4 weeks** . . .

	**All of the Time**	**Most of the Time**	**A Good Bit of the Time**	**Some of the Time**	**A Little of the Time**	**Noneof the Time**
a. Did you feel full of pep?	1	2	3	4	5	6
b. Have you been a very nervous person?	1	2	3	4	5	6
c. Have you felt so down in the dumps that nothing could cheer you up?	1	2	3	4	5	6
d. Have you felt calm and peaceful?	1	2	3	4	5	6
e. Did you have a lot of energy?	1	2	3	4	5	6

	All of the Time	Most of the Time	A Good Bit of the Time	Some of the Time	A Little of the Time	None of the Time
f. Have you felt downhearted and blue?	1	2	3	4	5	6
g. Did you feel worn out?	1	2	3	4	5	6
h. Have you been a happy person?	1	2	3	4	5	6
i. Did you feel tired?	1	2	3	4	5	6

**10. During the past 4 weeks, how much of the time has your physical health or emotional problems interfered with your social activities (like visiting with friends, relatives, etc.)? (Circle One Number)**	
All of the time	1
Most of the time	2
Some of the time	3
A little of the time	4
None of the time	5

11. How TRUE or FALSE is each of the following statements for you.(Circle One Number on Each Line)

	Definitely True	Mostly True	Don't Know	Mostly False	Definitely False
a. I seem to get sick a little easier than other people	1	2	3	4	5
b. I am as healthy as anybody I know	1	2	3	4	5
c. I expect my health to get worse	1	2	3	4	5
d. My health is excellent	1	2	3	4	5

Self-evaluation of negative symptoms (SNS)

Negative symptoms i.e. impairment of motivation for goal directed behavior, emotion and pleasure experience, social interest were measured using the Self-evaluation of Negative Symptoms(12). This scale evaluates 5 subdomains with 20 items corresponding to negative symptoms that characterize the negative syndromes of psychiatric syndrome: social withdrawal; diminished emotional range; alogia; avolition; anhedonia. It had been validated for assessing negative symptoms in schizophrenia suffering patients. The author S. Dolfus, gave its authorization to use the scale for the first time in our population. To our knowledge, this is the first time this scale is used in another population than patients with psychiatric disease. The subdomains are considered by scoring 4 items using a Lickert scale with a score from 0 to 2 corresponding to “0 = absolutely not agree, 1 = somewhat agree, 2 = strongly agree). Higher scores correspond to a more substantial presence of negative symptoms in each dimension and in total score(13).

Self-evaluation of Negative Symptoms

(SNS, S. Dollfus and C. Mach, V1_2014)

For each statement, put a cross in the box which best corresponds to your current feelings (based on the previous week).

	Strongly agree	Somewhat agree	Strongly disagree
1. I prefer to be alone in my corner			
2. I'm better off alone, because I feel uncomfortable when anyone is near me			
3. I'm not interested in going out with friends or family			
4. I don't particularly try to contact and meet friends (letters, telephone, text messaging, etc.)			
5. People say I'm not sad or happy and that I'm not often angry			
6. There are many happy or sad things in life but I don't feel concerned by them			
7. Watching a sad or happy film, reading or listening to a sad or happy story does not especially make me want to cry or laugh			
8. It is difficult for people to know how I feel			
9. I don't have as much to talk about as most people			
10. I find it 10 times harder to talk than most people do			
11. People often say that I don't talk much			
12. With friends and family, I want to talk about things but it doesn't come out			
13. I find it difficult to meet the objectives I set myself			
14. It's hard to stick to doing things on an everyday regular basis			
15. There are many things I don't do through lack of motivation or because I don't feel like it			

16. I know there are things I must do (get up or wash myself for example) but I have no energy			
17. I don't take any great pleasure in talking to people			
18. I find it hard to take pleasure even when doing things I have chosen to do			
19. When I imagine doing one thing or another, I don't feel any particular pleasure in the idea			
20. I am not interested in having sex			

Statistical analysis

Multivariate analysis:

We first conducted a descriptive analysis for socio-demographic variables and psychometric scale scores in groups categorized according to whether they reported an experience of fatigue. The normality of the data distribution was assessed using the Shapiro-Wilk test.

A univariate analysis was conducted for psychometric test scores between groups reporting fatigue (-F) and those not reporting fatigue (-NF), and within each diagnostic category (CD and UC). Means were compared using the unpaired t-student test, and in the event of non-normality of the data, the Mann-Whitney test was used. Contingency table tests were performed using the χ^2 test to test independence and association between variables; in the case of normality, a Fisher exact test was performed. Significance of results is defined by P value < 0.05.

Analysis of avolition scores taking into account personal history of depression was performed using a multi-way ANOVA (MANOVA) test, with alpha risk adjusted to 1%.

A multivariate analysis was performed with the variable Y= fatigue declared on the cohort involved dimensions X = "age category" ; "Personal history of depression" ; "Age of disease onset" ; "number of year lived with disease" ; "CFQ total score" ; "IBD Disk Energy score" ; "IBD Disk Stress / Anxiety score" ; "IBD DISK Total score" ; "SNS score Avolition" ; "SNS score Anhedonia" ; "HAD Anxiety score" ; "HAD Depression score" ; "SF 36 Energy/fatigue score" ; "CFQ psychological fatigue score" ; "CFQ physical fatigue score" ; "SNS Total score" ; "SF 36 physical functioning" ; "SF 36 Role limitation due to physical health" ; "SF 36 Role limitations due to emotional problems" ; "SF 36 emotional well being" ; "SF 36 Social functioning" ; "SF 36 Pain" ; "SF 36 General Health" ; "Covid total score".

Variables used regarding multivariable test are detailed in supplementary figure 1.

The statistical model of multiple logistic regression was used with the dependent variable "fatigue declaration". The independent variables were chosen according to their contribution to the PCA components in extreme quartiles: < -0.75 and >0.75 Graphic representations are presented in supplementary figure 1: "CFQ total score "; "SNS score Avolition"; "IBD DISK Total score"; "IBD Disk Stress / Anxiety score"; "CFQ physical fatigue score"; "HAD Anxiety score"; "SF 36 Pain"; "SF 36 Role limitation due to physical health"; "SF 36 Energy/fatigue score". Multicollinearity was tested and variables with a variance inflation factor (VIF) greater than 10 were excluded (“CFQ total score” VIF=15.28 and “CFQ physical fatigue score” VIF=15.27). The classification method used was the area under the ROC curve. The

Hosmer-Lemeshow test was applied to assess Goodness-of-fit.

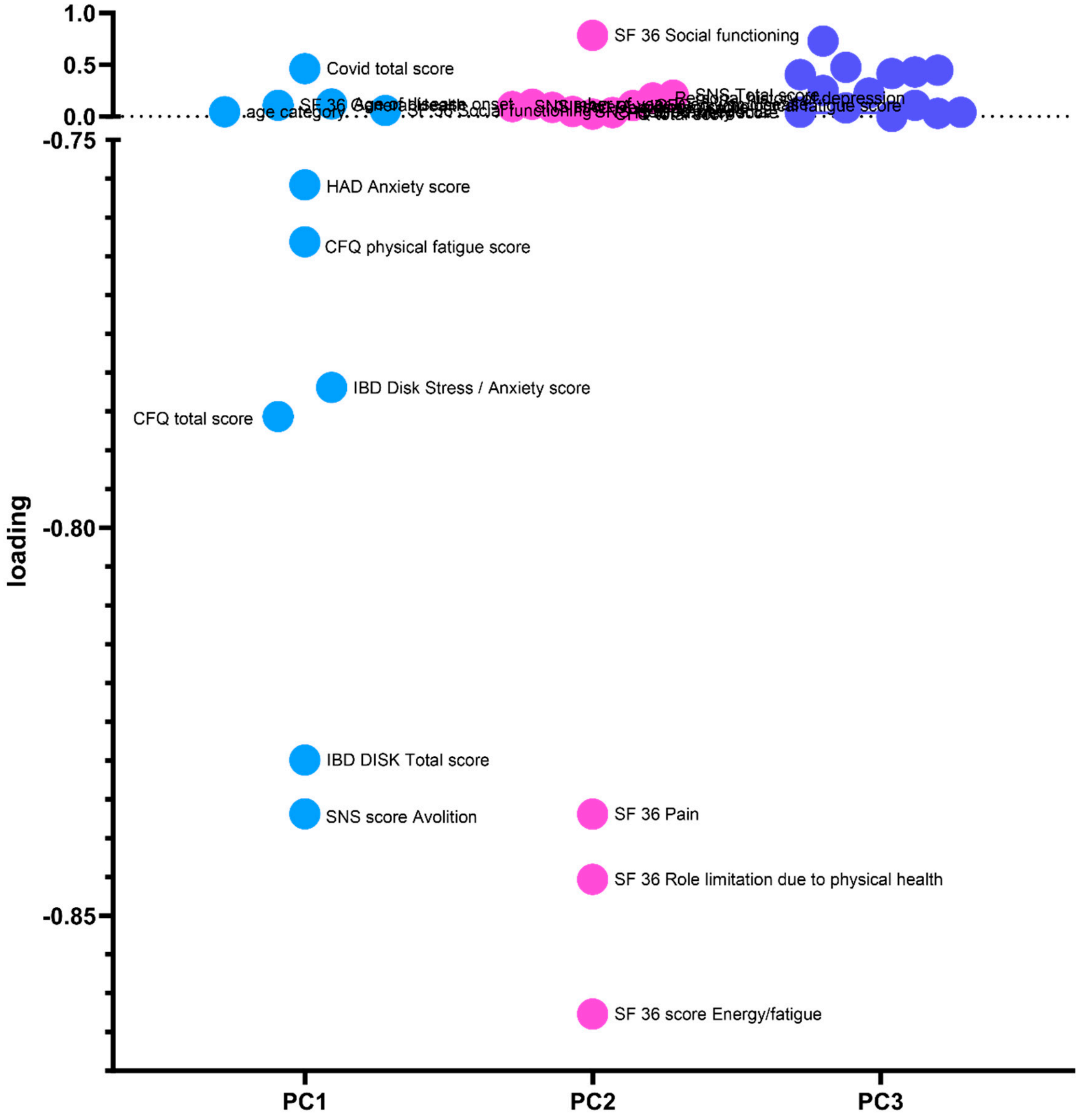

Correlation assessments were conducted using a two-tailed P-value Spearman nonparametric test. Missing data row were omitted (n=0). Results are expressed using r and it 95% confidence interval.

A simple logistic regression analysis was performed with the dependent variable "fatigue declaration" and as independent variable "SNS score Avolition". The null hypothesis was tested using a Likelihood ratio test (ratio = 16.85 (p=0.0001)). The area under the ROC curve was calculated, along with Tjur's R squared as a measure of goodness-of-fit (Tjur's R squared = 0.1064).

Statistical analysis and graphical representation were carried out using GraphPad Prism8 (San Diego, USA).

References :

1. Chalder T, Berelowitz G, Pawlikowska T, Watts L, Wessely S, Wright D, et al. Development of a fatigue scale. J Psychosom Res. févr 1993;37(2):147-53.

2. Jackson C. The Chalder Fatigue Scale (CFQ 11). Occup Med. janv 2015;65(1):86-86.

3. Cella M, Chalder T. Measuring fatigue in clinical and community settings. J Psychosom Res. juill 2010;69(1):17-22.

4. Le Berre C, Flamant M, Bouguen G, Siproudhis L, Dewitte M, Dib N, et al. VALIDation of the IBD-Disk Instrument for Assessing Disability in Inflammatory Bowel Diseases in a French Cohort: The VALIDate Study. J Crohns Colitis. 7 nov 2020;14(11):1512-23.

5. Colombel JF. Measuring Disability in IBD: The IBD Disability Index. Gastroenterol Hepatol. mai 2013;9(5):300-2.

6. Tannoury J, Nachury M, Martins C, Serrero M, Filippi J, Roblin X, et al. Determinants of IBD-related disability: a cross-sectional survey from the GETAID. Aliment Pharmacol Ther. mai 2021;53(10):1098-107.

7. Zigmond AS, Snaith RP. The Hospital Anxiety and Depression Scale. Acta Psychiatr Scand. 1983;67(6):361-70.

8. Pais-Ribeiro JL, Martins da Silva A, Vilhena E, Moreira I, Santos E, Mendonça D. The hospital anxiety and depression scale, in patients with multiple sclerosis. Neuropsychiatr Dis Treat. 22 nov 2018;14:3193-7.

9. Bell ML, Fairclough DL, Fiero MH, Butow PN. Handling missing items in the Hospital Anxiety and Depression Scale (HADS): a simulation study. BMC Res Notes. 22 oct 2016;9:479.

10. Brazier JE, Harper R, Jones NM, O'Cathain A, Thomas KJ, Usherwood T, et al. Validating the SF-36 health survey questionnaire: new outcome measure for primary care. BMJ. 18 juill 1992;305(6846):160-4.

11. Ware JE, work(s): CDSR. The MOS 36-Item Short-Form Health Survey (SF-36): I. Conceptual Framework and Item Selection. Med Care. 1992;30(6):473-83.

12. Dollfus S, Mucci A, Giordano GM, Bitter I, Austin SF, Delouche C, et al. European Validation of the Self-Evaluation of Negative Symptoms (SNS): A Large Multinational and Multicenter Study. Front Psychiatry. 31 janv 2022;13:826465.

13. Dollfus S, Mach C, Morello R. Self-Evaluation of Negative Symptoms: A Novel Tool to Assess Negative Symptoms. Schizophr Bull. mai 2016;42(3):571-8.
