# Supplementary material for: Avolition Characterizes the Chronic Fatigue Experienced in Quiescent Inflammatory Bowel Disease
Source: Biomedicines. 2025 Jan 7;13(1):125. doi: 10.3390/biomedicines13010125 (PMC11761293; doi:10.3390/biomedicines13010125)
Supplement: Supplementary file 1 [file biomedicines-13-00125-s001.zip › Supplementary Table S1.pdf]

cohort characteristic

No previous history		CD-F	CD-NF	CD	UC-F	UC-NF	UC
of depressive disorder	n	21	22	43	19	21	40
	%	67.7	75.9	71.7	70.4	91.3	80.0
Previous history		CD-F	CD-NF	CD	UC-F	UC-NF	UC
of depressive disorder	n	10	7	17	8	2	10
	%	32.3	24.1	28.3	29.6	8.7	20.0

Avolition score

No previous history of depressive disorder		CD-F	CD-NF	p
	n	21	22	0.0277
	mean	3.19	1.816	
	SD	2.064	1.622	
		UC-F	UC-NF	
	n	19	21	0.0044
	mean	2.737	0.408	
	SD	2.423	1.411	
Previous history of depressive disorder		CD-F	CD-NF	
	n	10	7	0.0516
	mean	5.1	3	
	SD	2.234	1.633	
		UC-F	UC-NF	
	n	8	2	0.53
	mean	3	4	
	SD	1.773	2.828	

Supplementary Table S1 : Description of cohort characteristics regarding previous history of depressive disorder. CD: Crohn’s disease, UC: ulcerative colitis, -F : declaration of fatigue experience, -NF : no declaration of fatigue experience, SD: standard deviation.
