# Supplementary material for: Avolition Characterizes the Chronic Fatigue Experienced in Quiescent Inflammatory Bowel Disease
Source: Biomedicines. 2025 Jan 7;13(1):125. doi: 10.3390/biomedicines13010125 (PMC11761293; doi:10.3390/biomedicines13010125)
Supplement: Supplementary file 1 [file biomedicines-13-00125-s001.zip › supplementary Table S2.pdf]

Multivariate analysis	Dependent variable (Y)	Independent variables (X)
PCA	Fatigue declaration (no/yes)	Age category
		Personal history of depression
		Age of disease onset
		Number of years lived with disease
		CFQ total score
		CFQ psychological fatigue score
		CFQ physical fatigue score
		IBD Disk Energy score
		IBD Disk Stress / Anxiety score
		IBD Disk Total score
		SNS score Avolition
		SNS score Anhedonia
		SNS Total score
		HADS Anxiety score
		HADS Depression score
		SF 36 score Energy/fatigue
		SF 36 physical functioning
		SF 36 Role limitation due to physical health
		SF 36 Role limitations due to emotional problems
		SF 36 emotional well being
		SF 36 Social functioning
		SF 36 Pain
		SF 36 General Health
		Covid total score
Multiple logistic regression	Fatigue declaration (no/yes)	CFQ Score continu
		CFQ physical fatigue score
		SNS score Avolition
		IBD Disk Total score
		IBD Disk Stress / Anxiety score
		HADS Anxiety score
		SF 36 score Energy/fatigue
		SF 36 Role limitation due to physical health
		SF 36 Pain
Spearman correlation	CFQ total score (continuous)	SNS score Avolition
		IBD Disk Total score
		IBD Disk Stress / Anxiety score
		HADS Anxiety score

Supplementary Table S2: description of variables involved in the multivariable statistical analysis. (PCA: principal component analysis, CFQ : Chalder's Fatigue Questionnaire, SNS : self evaluation of negative symptoms, HADS : hospital anxiety and depression scale, SF 36 : short Form 36,
