# Supplementary figures and images for: Avolition Characterizes the Chronic Fatigue Experienced in Quiescent Inflammatory Bowel Disease
Source: Biomedicines. 2025 Jan 7;13(1):125. doi: 10.3390/biomedicines13010125 (PMC11761293; doi:10.3390/biomedicines13010125)

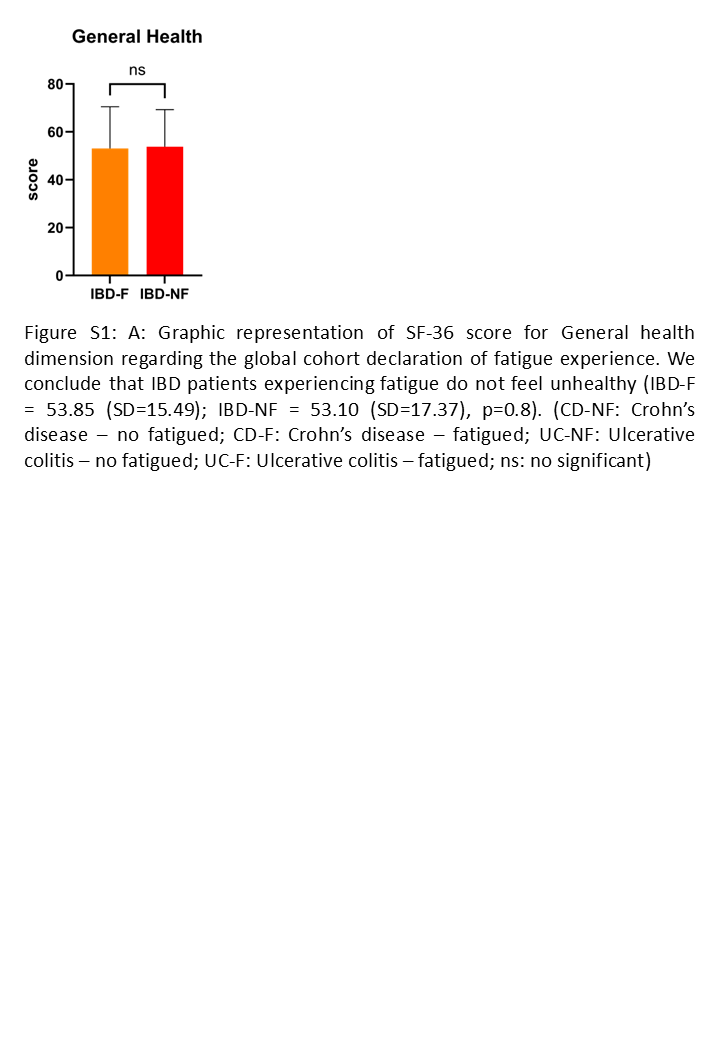

Supplement: Supplementary file 1 [file biomedicines-13-00125-s001.zip › Figure S1.tif]

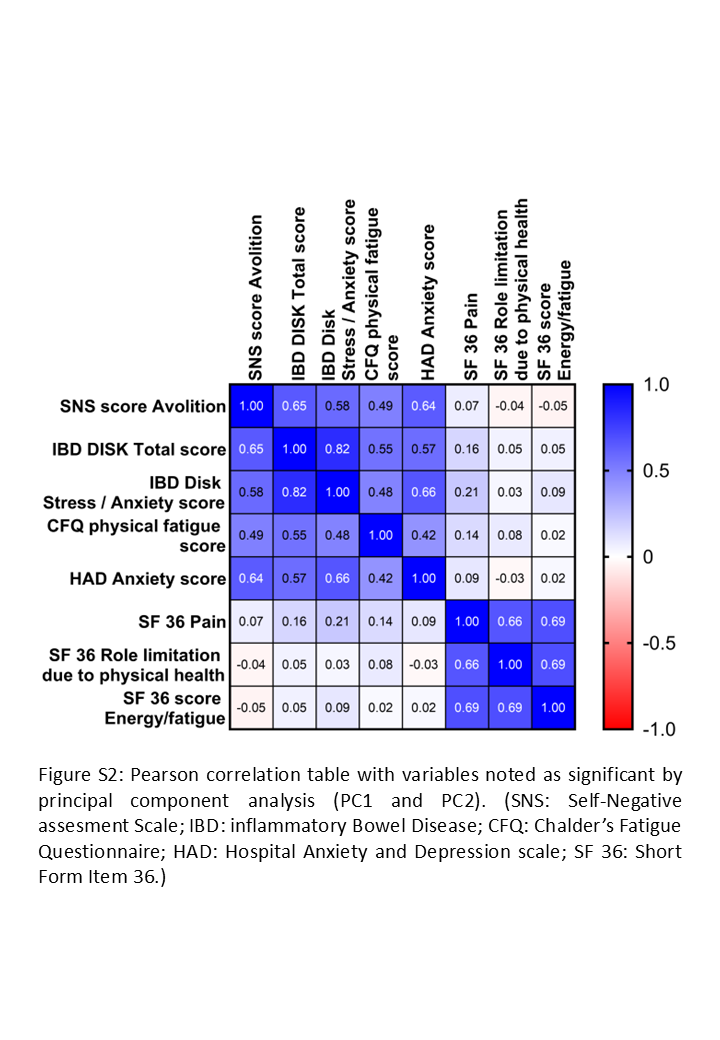

Supplement: Supplementary file 1 [file biomedicines-13-00125-s001.zip › Figure S2.tif]
